# Physical and Chemical Modifications of Polymeric Surfaces to Enhance Epithelial Cell Adhesion

**DOI:** 10.1021/acsomega.5c06437

**Published:** 2025-10-29

**Authors:** Laura M. S. Santos, Jonathas M. Oliveira, Artur F. Sonsin, Sendy M. S. Nascimento, Vitor M. L. Fonseca, Juliane P. Silva, Cléber R. Mendonça, Alcenísio J. Jesus-Silva, Emiliano Barreto, Eduardo J. S. Fonseca

**Affiliations:** † Optics and Nanoscopy Group, Institute of Physics, 28112 Federal University of Alagoas (UFAL), 57072-970 Maceio, Alagoas, Brazil; ‡ Federal Institute of Alagoas (IFAL), 57230-000 Coruripe, Alagoas, Brazil; § Laboratory of Cell Biology, Institute of Biological Sciences and Health, Federal University of Alagoas (ICBS/UFAL), 57072-970 Maceio, Alagoas, Brazil; ∥ Federal University of Rondônia (UNIR), 76940-000 Rolim de Moura, Rondônia, Brazil; ⊥ Institute of Physics of São Carlos, University of São Paulo, CP 369, 13560-970 São Carlos, São Paulo, Brazil

## Abstract

In tissue engineering, 3D scaffolds and chemical treatments are often used to provide a cell-friendly surface that improves cell adhesion and tissue growth. Indeed, cell adhesion can be regulated through physicochemical modifications of the substrate surface, including wettability, surface charge, and roughness. In this work, we present the synthesis, characterization, and cytocompatibility analysis of photoresins suitable for fabricating cell scaffolds via two-photon polymerization. Furthermore, we demonstrate a simple surface treatment method that promotes cell adhesion. This method alters the surface charge of the polymer and enhances the adhesion of epithelial cells. Our results indicate an efficient approach to modifying the surface of biocompatible polymer scaffolds with the purpose of enhancing the performance of cell functions that are suitable for tissue engineering and regenerative medicine.

## Graphical abstract

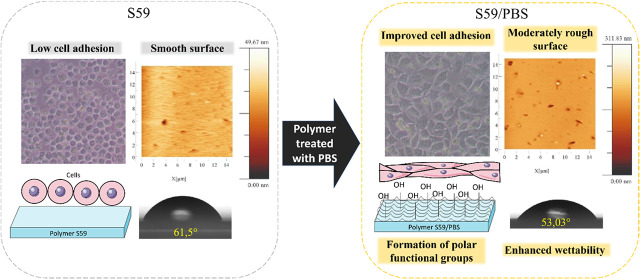

## 1 Introduction

Polymeric materials are at the forefront of the development of biophotonic and biomedical devices. These materials have numerous applications, including biosensors for diagnosis, implants, and 3D environments suitable for cell culture.^1–3^ The attractive features of polymers include ease of synthesis, large-scale production, capacity for functionalization with nanoparticles and other chemical functional groups, as well as their interesting optical and mechanical properties.^4,5^ Furthermore, they can be synthesized to exhibit adjustable flexibility, which makes them an excellent tool for drug delivery and scaffolds for cell and tissue growth.^6–9^ In particular, acrylic-based polymers are widely used in biomedical applications due to their biocompatibility, biodegradability, and mechanical properties.^10–13^

Cell adhesion to a surface is crucial for various cellular functions, including proliferation and differentiation. It also plays a significant role in the development of biomaterials and implantable sensors or devices.^14^ Understanding the factors that affect the cell-surface adhesion allows for the control of cell behavior in the local environment, which benefits research in tissue engineering technologies.^15^ Indeed, the relationship between cellular behavior and the wettability of polymer surfaces has been investigated in the literature, particularly in relation to cell adherence.^16^ Although studies have suggested an improvement in cell adhesion on hydrophilic surfaces, there are other factors that must be considered for a complete understanding of cell behavior, such as surface energy, roughness, and porosity of polymer surfaces.^17–20^

For instance, Majhy and colleagues^21^ showed that cervical and breast cancer cell lines exhibit different behaviors on the same substrate, adjusting their adherent properties to different roughness ratios. This demonstrates that each cell lineage responds in a particular way to a specific feature of the substrate. Indeed, in cell-material interactions, both physical and chemical features need to be adjusted to create a favorable environment for the cells.^22^

Numerous approaches for surface modification have been used to alter the properties of materials and enhance cell attachment, including plasma treatment, chemical vapor deposition, dynamic surface modification, protein adsorption, and silanization.^23,24^ Despite the great diversity of methods capable of inducing polymer surface modification,^25–27^ there is still a need to propose cheaper and faster methods for surface treatment. Furthermore, it is important to ensure that the biocompatibility of each new polymeric material is accompanied by a suitable surface treatment. This approach helps create a surface that is more biologically compatible with the organism.

Following an eco-friendly trend, the use of phosphate-buffered saline (PBS) solution to induce surface modifications in inorganic materials meets current requirements by facilitating a reduction in the use of organic reagents and minimizing waste production.^28,29^ PBS is commonly used to simulate biological solutions in research and laboratory settings. It is chosen for its ability to closely match the osmolarity and ion concentrations found in the human body. As an isotonic and nontoxic medium, PBS provides a suitable environment for maintaining the viability and functionality of living cells.

While the technique of increasing the surface hydrophilicity of polymers using PBS is well-documented,^30–36^ the effects of PBS on the roughness and functional group composition of polymer surfaces based on pentaerythritol triacrylate (here referred to as P59) and trimethylolpropane ethoxylate triacrylate (here referred to as S59) in the context of creating a cell-friendly surface have not been extensively explored or described, to the best of our knowledge.

In this work, we conducted an evaluation of the adhesion of human epithelial A549 cells on acrylate-based photoresins. We further investigated the impact of chemical modification induced by PBS on cell adhesion to polymeric surfaces. Our results were supported by several characterization methods.

## 2 Material and Methods

### 2.1 Materials

The monomers used in the preparation of photoresins were pentaerythritol triacrylate and trimethylolpropane ethoxylate triacrylate. The photoinitiator 2-hydroxy-4′-(2-hydroxyethoxy)-2-methylpropiophenone (Irgacure 2959) was used to initiate photochemical cross-linking. All chemicals were purchased from Sigma-Aldrich.

### 2.2 Preparation and Mechanical Characterization of the Polymeric Substrates

Two distinct polymeric resins were prepared by combining a monomer with 1 wt % of the photoinitiator Irgacure 2959. The first formulation, referred to as P59, was based on monomer pentaerythritol triacrylate, while the second, named S59, was based on monomer trimethylolpropane ethoxylate triacrylate. The photoresins were gently mixed for 24 h at room temperature.

The substrates were molded into circular discs (16 mm in diameter × 0.6 mm in thickness) and polymerized under UV light for 20 min. Afterward, the samples were carefully immersed in ethanol for 20 min to wash away the unpolymerized resin. This process was repeated three times to ensure complete removal.

A strategy to make substrates more hydrophilic and potentially more adhesive to cells was to treat the polymeric surfaces with phosphate-buffered saline (PBS 1×, pH 7.4). A PBS solution consisting of NaCl, KH_2_PO_4_ and Na_2_HPO_4_ was prepared. Subsequently, the polymeric substrates were treated for 60 min at room temperature. Here, the control consisted of commercial adherent cell culture plates designed to promote cell adhesion. Finally, the samples were dried in an oven at 50 °C for 10 min. After the PBS treatment, the samples were renamed P59/PBS and S59/PBS.

The mechanical properties of the polymers were evaluated by the Three-Point Flexural Strength Test^37^ using the Microtensile OM100 semiuniversal testing machine (Odeme Dental Research, SC, BRA). The test specimens were prepared for each experimental group (n = 5) in a stainless-steel matrix (2 mm × 2 mm × 25 mm) and after 20 min of UV-lamp polymerization they were washed three times with ethanol (99.8% PA, Neon). The 3-bending point testing was conducted at room temperature with a loading ratio of 50 N/min and a speed of 0.75 ± 0.25 mm/min, until the sample fractured and the resulting stress–strain curve was registered. The Elasticity modulus for bending (*E*_*B*_) is related to

1 $$\sigma =E_{B}\varepsilon$$

where σ is the Flexural Stress (MPa) and ε is the Flexural Strain (mm/mm). The modulus of elasticity (*E*_B_) is calculated by the tangent, within the elastic limit, of the stress–strain curve.

### 2.3 Contact Angle Measurements

The investigations of wetting phenomena on the substrate surface were made using the sessile drop method, an accurate and reproducible optical method for contact angle measurements. Only one drop of water (3 μL) was deposited on different samples. The contact angle measurement was performed using Theta Optical Tensiometer (T200 Biolin Scientific) with an embedded CCD camera and Attension software. For wetting phenomena, all five measurements for each sample were carried out at 23 °C.

The interactions between solid and liquid are important in various processes as they determine the adhesion between the phases. Solid–liquid interactions are determined by the surface free energy (SFE) of the solid and the surface tension of the liquid applied. The SFE method used in this work was the Owens-Wendt-Rabel-Kaelble model, which uses a geometric mean to treat the molecular interactions. The idea of this model is to divide the SFE into individual components. We considered only polar interactions with films. The polar liquid chosen was water due to its large polar component, availability, and nontoxic nature. The SFE polar component (γ_*p*_) was calculated using the equation given below:

2 $$\gamma_{p}=0.5\cdot \gamma_{\mathrm{lv}}\left(1+\cos\theta \right)$$

where γ_*lv*_ is the surface tension of the polar liquid, in this case, water (72.8 mJ/m^2^) and θ is the angle formed in the contact angle measurement.

### 2.4 Atomic Force Microscopy (AFM)

All measurements were obtained using a standard AFM setup (Multiview 4000, Nanonics, Israel), with a combined optical microscope (BXFM, Olympus, Japan). The AFM system was acoustically isolated to reduce any interference from ambient noise during the measurements, and the instrument was secured on an active damping table to suppress mechanical noise. The topography of the substrates was imaged (256 × 256 pixels) in tapping mode with a scan rate of 0.3–1.0 Hz in an area of 15 × 15 μm^2^. About nine regions were analyzed for each substrate. The roughness of the Control (CTRL), S59, P59 and PBS-treated samples was analyzed using WSxM software, through the mean parameters of the roughness of each sample. For each image, two areas of 7.5 × 7.5 μm^2^ were selected, and the average roughness (*R*_*a*_) was calculated as the absolute mean of the heights of the irregularities along the profile.

3 $$R_{a}=\frac{1}{N}\sum_{i=1}^{N}\left|z_{i}-\mathrm{z̅}\right|$$

where *N* is the number of sample points, *z*_*i*_ is the height of each sample point, and z̅ is the average height of the sample points.

Data were expressed as mean ± standard deviation (SD). To show that all data were normally distributed, the Kolmogorov–Smirnov and Shapiro-Wilk tests were performed at the 0.05 level, assuming a normal population.

Furthermore, three additional parameters, skewness, kurtosis, and average height, were analyzed to quantify the topography of the S59 and P59 polymer surfaces subjected to PBS treatment. Skewness measures the symmetry of the height distribution relative to the mean; positive skewness values indicate a greater presence of peaks than valleys on the surface, with sharper protrusions, whereas negative skewness values correspond to surfaces with more valleys exhibiting deeper depressions.^38^ This parameter can help determine whether the surface possesses features that favor cell adhesion, such as valleys for anchoring, or peaks that hinder interaction.^16,19,25^ Kurtosis describes the sharpness of the height distribution; in practical terms, high kurtosis values indicate the presence of sharp peaks or deep valleys.^39^

### 2.5 Fourier Transform Infrared (FTIR) and UV–Vis Spectroscopies

The infrared spectra (FTIR) of the samples were obtained at room temperature using an IRPrestige-21 spectrophotometer (Shimadzu, Kyoto, Japan) coupled with an attenuated total reflectance (ATR) accessory with ZnSe crystal. The spectral range was 4000–800 cm^–1^, with a resolution of 4 cm^–1^ and 120 scans. The band intensities were expressed in transmittance (%) with a diffuse reflectance accessory (DRS-8000). The FTIR spectra of each sample were obtained before and after the PBS treatment. The absorption spectra of the samples were recorded using a UV-3600 spectrophotometer (Shimadzu, Kyoto, Japan). The spectra were recorded in the range of 300–700 nm.

### 2.6 Cell Culture

Human alveolar epithelial cell line A549 was used in this study and grown in Dulbecco’s Modified Eagle’s Medium (DMEM) supplemented with 10% fetal bovine serum (FBS), 1.467% l-glutamine, 1.4% glucose, 1% sodium pyruvate, and 0.02% penicillin/streptomycin, and maintained at 37 °C in a 5% CO_2_ humidified atmosphere. Cells were passaged every 2 days, with the medium being changed.

Prior to cell adhesion studies with the polymers of interest, the culture wells were covered with 1 mL of polymeric substrates or PBS. After 1 h, the excess liquid was withdrawn, and the residual humidity was dried in a heated incubator for 5 min. Next, the 12-well plate was transferred to a laminar flow hood for 1 h under UV light.

### 2.7 Cell Seeding and Adhesion

Cells were detached from their culture dishes by incubating with a solution of 2.5 g/L trypsin and 0.38 g/L of ethylenediaminetetraacetic acid (EDTA) for 5 min. The dissociated cells were seeded (3 × 10^4^ cells/well) on the polymer surface and maintained in supplemented DMEM medium under culture conditions for 24 h. Then, the cells were washed twice with DMEM medium to remove nonadhered cells, and photographs were taken at 0 and 24 h to evaluate the morphological differences related to adhesion.

### 2.8 Quantification of Cell Adhesion

The interaction of epithelial cells after contact with polymeric substrates, before and after PBS treatment, was observed by optical microscopy (100× magnification). Images of the cells were captured in two different regions of the substrate. In each image, we selected four regions with the same area (∼57 × 57 μm^2^). For each specific region, the cells were counted, and their morphology was analyzed using Gwyddion software (version 2.3), and the resulting data were processed with Origin (OriginLab). The cell adhesion quantification was guided by the morphological aspects of cells evoked by adhesive behavior after interaction with polymeric substrates. We considered that nonadherent cells present a rounded and shiny appearance, while adhered cells appear more elongated with spindle-shaped, fibroblast-like morphology (Figure 1).

**Figure 1:**
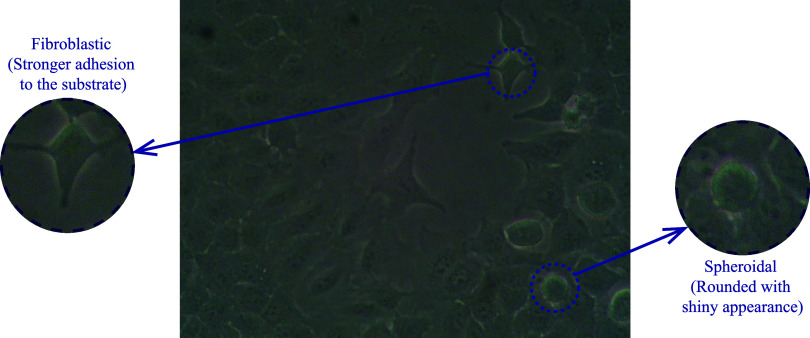
Light microscope images of A549 cells grown in DMEM medium for 24 h on commercial adherent cell culture plates (100× magnification). Cells identified by arrows show spheroidal or fibroblast-like morphology and are enlarged in the inset images.

Quantitative analysis of the morphology was carried out for a better understanding of the changes in adhesion-induced cell shape. Parameters such as Shape Factor and cellular aspect ratio were calculated. The Shape Factor ϕ (ranging from 0 to 1) represents the ratio between the cell surface area and the cell perimeter.^40,41^ The cellular aspect ratio is approximately the ratio of the minor axis (width) to the major axis (length) of an ellipse fitted to the cell.^42^ Therefore, through these parameters, it was possible to indicate how circular the cells are. This means that, for more rounded cells, the area-perimeter ratio increases, as well as the relationship between cell axes, making the Shape Factor and cellular aspect ratio closer to 1. On the other hand, for cells that are adhered to the substrate and acquire elongated geometries, these parameters are less than 1. Cell morphological parameters were calculated for at least 60 manually labeled cells for each experimental condition. Experimental data were expressed as mean ± standard deviation.

## 3 Results

The characterization of the mechanical behavior of polymers is an important parameter for determining their potential applications in biological contexts. Figure 2 shows the results of the three-point bending tests for the P59 and S59 polymers.

**Figure 2:**
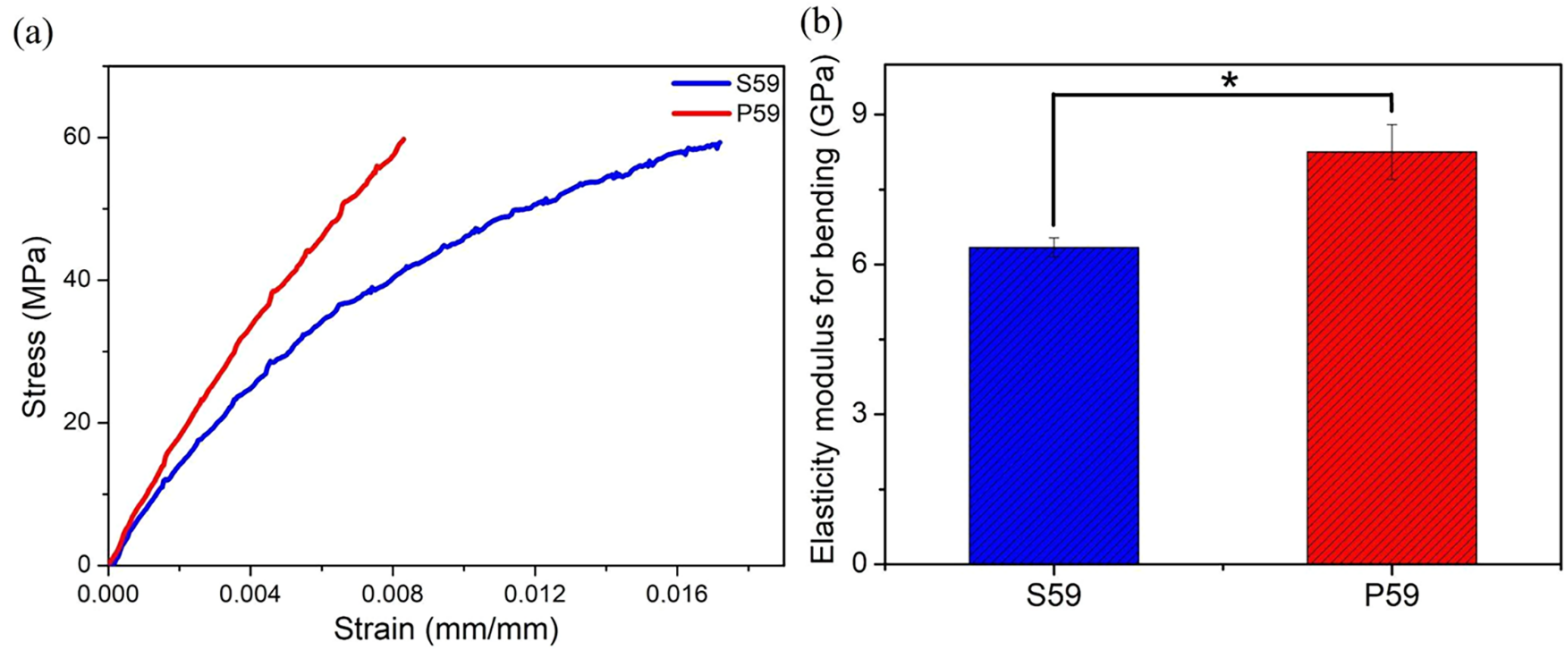
Mechanical analysis of P59 and S59 polymers (a) Strain–Stress curve and (b) Statical analysis of elastic modulus. One-Way ANOVA analysis. *n* = 5; Significantly different when *p* value <0,01. * *p* value = 0.006.

Figure 2a shows the flexural stress–strain curves. Based on the analysis of these curves, the P59 polymers exhibited an elastic modulus of 8.25 ± 0.05 GPa, whereas the S59 polymers showed a significant reduction in the elastic modulus to 6.34 ± 0.05 GPa, with the corresponding values shown in Figure 2b. This difference is statistically significant, with *p*-values of 0.006. The statistical data, analyzed with a significance level of *p* < 0.01, suggest that the marked increase in elongation at break and the reduction in the elastic modulus of the S59 polymers indicate less rigid, more flexible, and more resilient matrices.

As shown in Figure 3, the polymers P59 (pentaerythritol triacrylate) and S59 (trimethylolpropane ethoxylate triacrylate) were analyzed using UV–vis spectroscopy. The characteristic features of the UV–vis absorbance spectra revealed that the onset of absorption was identical prior to polymerization. Irgacure 2959 was responsible for the predominant absorption band below 350 nm and the transparency to visible radiation in both samples (Figure 3).

**Figure 3:**
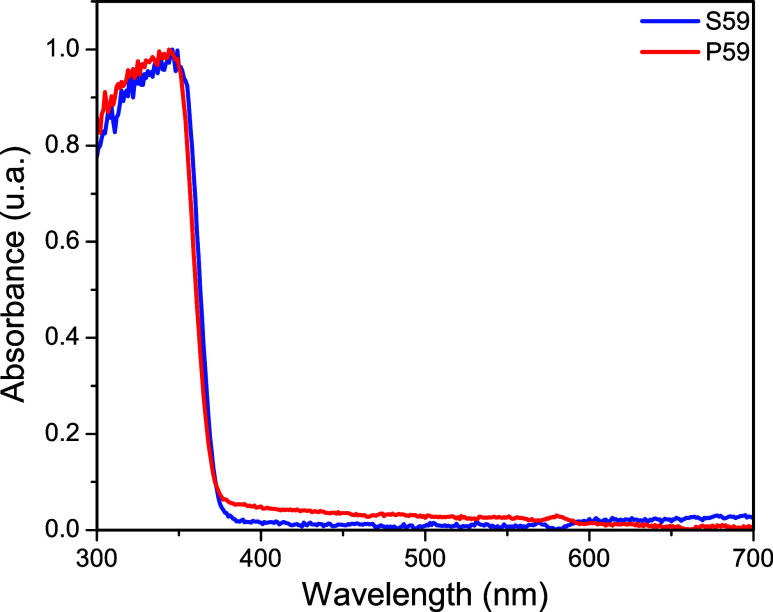
UV–visible absorption spectrum of the polymeric samples P59 and S59.

From this point forward, P59 and S59 were used as substrates to promote cell adhesion. For this purpose, cells in a fibroblastic (elongated) or rounded shape after 24 h of exposure to polymeric surfaces were quantified. In addition, in another set of experiments, we used PBS to induce changes on the surface of polymers P59 and S59 before allowing cells to interact. Figure 4a shows the percentage of adherent cells after interaction with each sample, treated or not with PBS.

**Figure 4:**
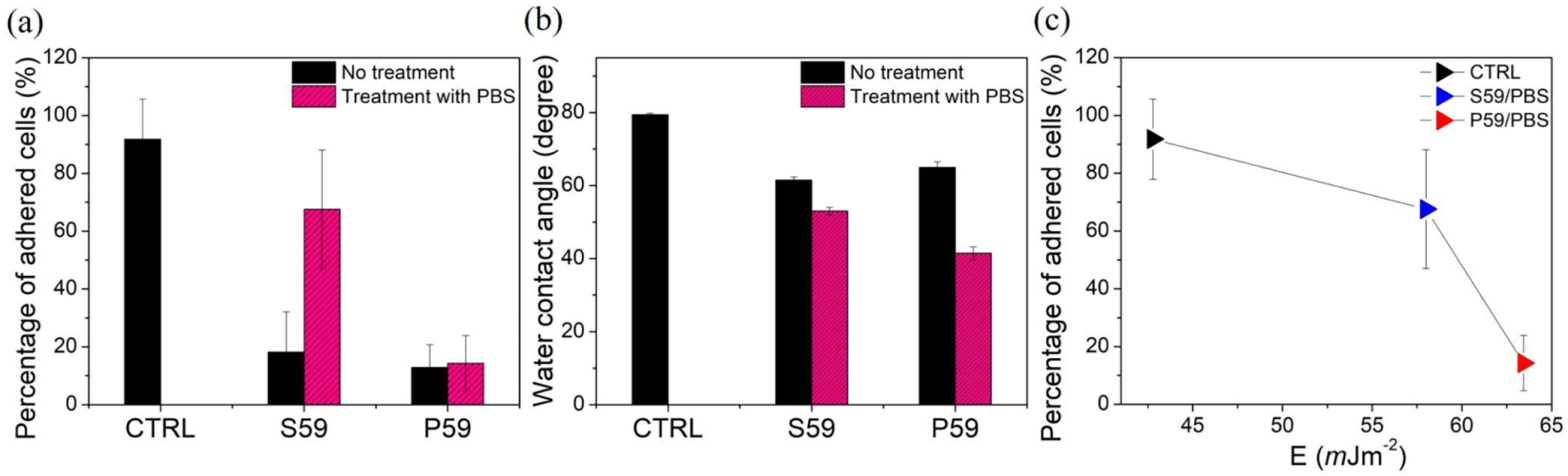
Evaluation of the adhesive behavior of cells (a), wettability measurements (b), and surface free energy (c) of polymeric surfaces treated or not with PBS.

By observing the adhesion of cells and comparing them with the control plates, it was possible to note that S59 and P59 did not present enough physicochemical features to promote cell adhesion. After treatment with PBS on the surface of the samples, the S59/PBS substrate allowed an increase in the adhesive behavior of cells by about 3 times compared to the condition without treatment with PBS (Figure 4a). The cell behavior on S59 substrates after PBS treatment was comparable to the behavior observed on commercial control plates. The P59 polymer did not induce changes in cell adhesive behavior after PBS treatment (Figure 4a). These results revealed that S59 substrates, after PBS treatment, showed a cell-friendly surface for the adhesion of epithelial cells. Although our study focused on the initial events of cell adhesion in response to PBS-induced surface modification, future investigations will provide deeper insight into the long-term behavior of cells on treated polymeric substrates and help clarify the mechanisms underlying enhanced adhesion.

Figure 4b shows the results for water wettability measurements, conducted before and after the PBS treatment. The contact angle of all samples exhibited a reduction of approximately 20%, indicating an increase in hydrophilicity due to the PBS treatment (Figure 4b). Notably, the P59 samples demonstrated a greater decrease in contact angle, shifting from 64.93 to 41.48° following the PBS treatment (Figure 4b).

The surface free energy was calculated via eq 2 and related to the cell adhesion in Figure 4c. We can observe that there was a correspondence between the free energy and the cell binding to the substrate. It is important to note that the control (CTRL) substrates used in this study are commercial polymeric plates treated for promoting cell adhesion. For surface free energy (SFE) values close to the CTRL substrate, *E* = 42.78 mJ/m^2^, the percentage of adhesion increases. Therefore, cell adhesion is higher on the S59/PBS substrate compared to the P59/PBS substrate.

The polar component of SFE showed a correspondence with cell adhesion. The CTRL substrate had the lowest polar SFE value, indicating the best cell adherence. The S59 PBS-treated substrate had a higher polar SFE of approximately 57 mJ/m^2^, which also promoted cell adhesion. However, a larger SFE (64 mJ/m^2^), as observed in the P59 PBS-treated substrate, resulted in lower cell adhesion. These results suggest that the polar SFE plays a significant role in cellular adhesion.

As the topography of a surface is an important environmental component able to influence cell adhesion, we focused on evaluating the roughness of the polymeric surfaces treated or not with phosphate-buffered saline (PBS) to regulate cell adhesion. To achieve this objective, the AFM system was used to observe the topographies of the polymeric substrates, allowing for the investigation of surface nanoroughness. Figure 5a shows the average roughness (*R*_*a*_) of the polymeric substrates before and after PBS treatment.

**Figure 5:**
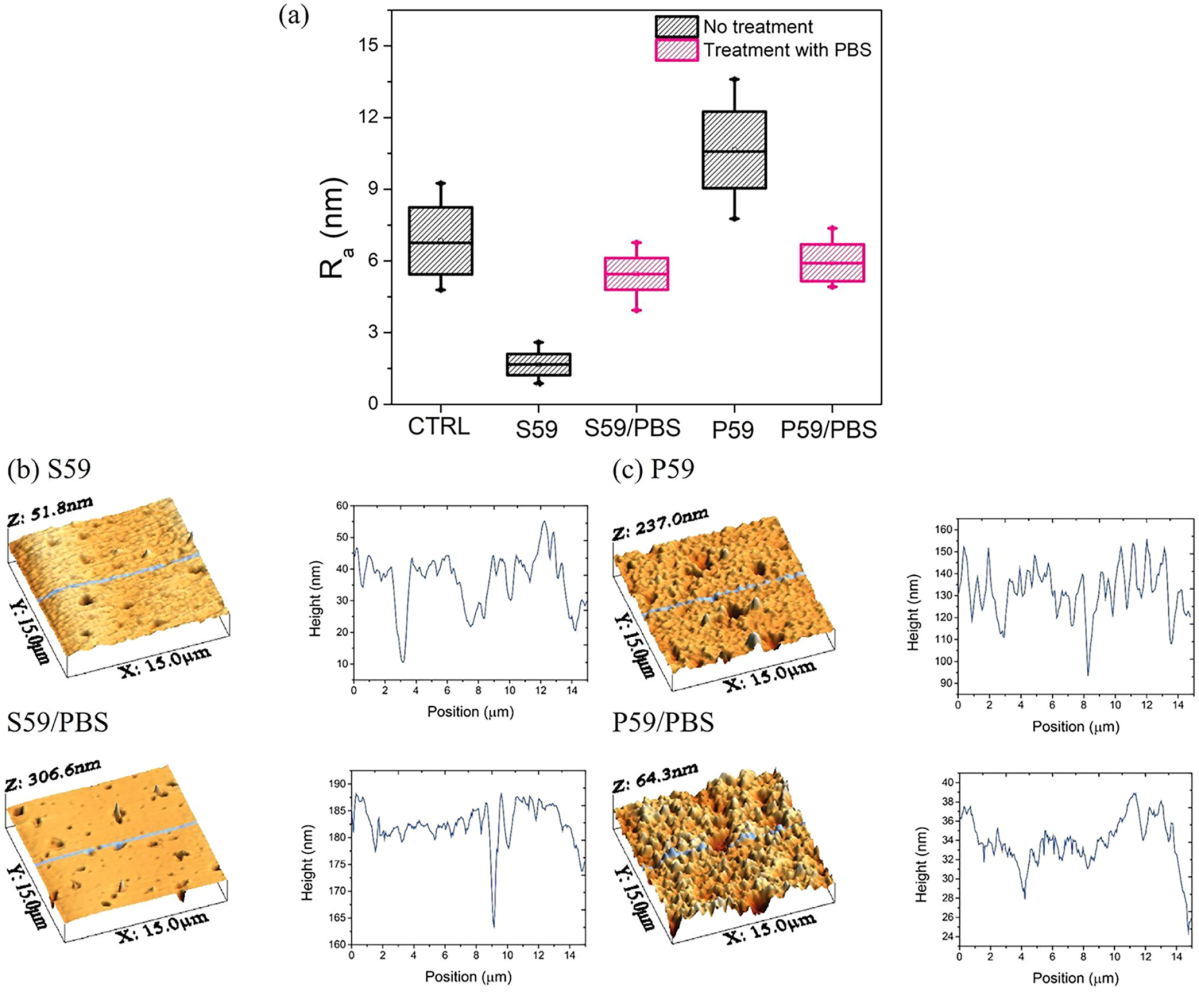
(a) The average roughness (*R*_*a*_) values of the CTRL, S59, and P59 samples, and those treated with PBS. AFM topographic images obtained through atomic force microscopy of the sample substrates, namely (b) S59, S59/PBS, (c) P59, and P59/PBS.

As shown in Figure 5a, the average roughness of all samples was modified by the PBS treatment, making P59 samples less rugged while increasing the roughness of the S59 samples. Interestingly, the PBS buffer adjusted the average roughness of all surfaces to approximately 5.30 nm. The normality test showed that the population means are significantly different at the 0.05 level.

We analyzed a transverse cut of the polymeric planes (Figure 5b,c). In these figures, we can observe that the S59/PBS substrate is notably less wrinkled than the P59/PBS samples. Thus, the surface roughness ratio (*r*), defined as the ratio between the actual surface area and the projected solid surface area, can provide more accurate insights into the behavior of the cells on each substrate.

Indeed, Figure 6 shows the relationship between the roughness ratio (*r*) and the percentage of adherent cells on each substrate. We can observe that the CTRL samples present an interesting roughness profile for cell behavior, with *r* = 1.0075. This topography pattern was also observed in the S59/PBS samples, with *r* = 1.0052, such that the cell count resembled that of seeded adherent cells on the CTRL substrates. However, in the P59/PBS samples, *r* = 1.0138, making the topography sufficiently wrinkled to discourage cell adhesion. It is important to keep in mind that the roughness ratio is just a reference number, representing a convergence point between a specific cell and a particular nanotopography. Nevertheless, the roughness ratio (*r*) should be considered a more significant parameter than the average roughness (*R*_*a*_), as it quantifies the nanowrinkling of the surface. Indeed, the cellular behavior in response to the nanoroughness of the substrate can be expressed by its capacity for adhesion to the substrate and subsequent morphological change.

**Figure 6:**
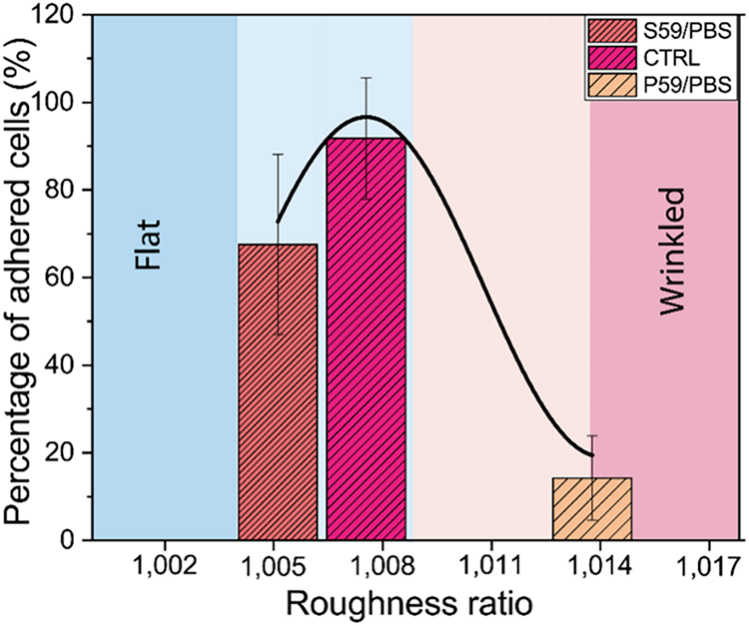
Effect of the roughness ratio of polymeric films treated with PBS on the adhesion behavior of A549 cells.

The investigation of the skewness, kurtosis, and average height parameters, presented in Figure 7, provides a more detailed assessment of the influence of PBS treatment on the topography of the S59 and P59 polymer surfaces. Figure 7a shows that the CTRL and S59 samples exhibit mean heights close to zero, with narrow and symmetric distributions (particularly in S59), suggesting more uniform surfaces. In contrast, S59/PBS displays the lowest mean height (most negative) and the widest dispersion, including negative outliers (deep valleys), indicating that PBS treatment in S59 increased the presence of surface depressions, resulting in a more complex and potentially functional topography. The P59 and P59/PBS samples exhibit moderately negative mean heights with greater variability, with P59/PBS presenting positive outliers, suggesting a surface topography characterized by prominent peaks or surface discontinuities.

**Figure 7:**
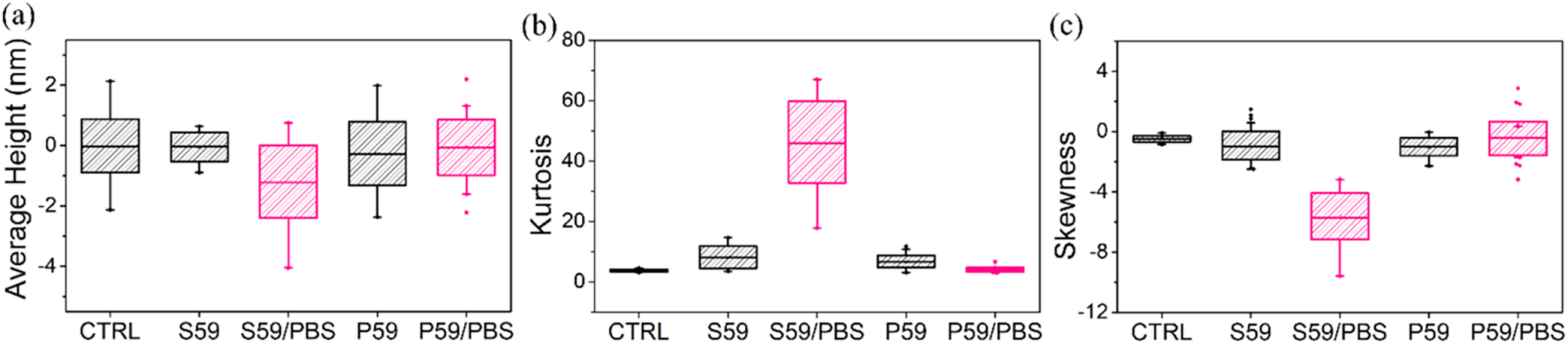
Parameters of (a) average height, (b) kurtosis, and (c) skewness characterizing the topography of S59 and P59 polymer surfaces and PBS-treated samples.

The investigation of kurtosis, shown in Figure 7b, demonstrated that CTRL presents a low kurtosis value, close to that of a normal distribution, indicating a balanced surface without many extremes. S59 and P59 polymers exhibit slightly higher kurtosis values, suggesting the emergence of more defined peaks and valleys compared to CTRL. The P59/PBS polymer displays a lower kurtosis, whereas S59/PBS exhibits an extremely high kurtosis, indicating a surface with a high density of extreme features, including sharp peaks and deep valleys.

The analysis of skewness values, presented in Figure 7c, revealed that CTRL exhibits values close to zero, indicating a symmetrical distribution between peaks and valleys. S59 and P59 polymers showed negative skewness values, indicating a predominance of valleys. In the P59/PBS samples, skewness was even closer to zero, but with positive outliers, indicating the presence of occasional prominent peaks. In contrast, S59/PBS displayed markedly negative values, around −6, and a wide range, indicating a surface dominated by deep valleys after PBS treatment.

Next, infrared spectroscopy was employed to identify the chemical changes occurring in the functional groups of the polymeric substrates treated with PBS. Figure 8 shows the recorded FTIR spectra of untreated polymeric samples (black lines), PBS-treated samples (blue lines), the PBS buffer sample (dark yellow lines), and the difference spectrum (pink lines) between the surfaces before and after PBS treatment. It is evident that the S59/PBS samples were affected by the phosphate buffer treatment, unlike the P59/PBS samples. Observing the pink lines (Figure 8a,b), we can identify some important vibrational bands excited by the PBS treatment on the S59 substrate that were not observed on the P59 substrate.

**Figure 8:**
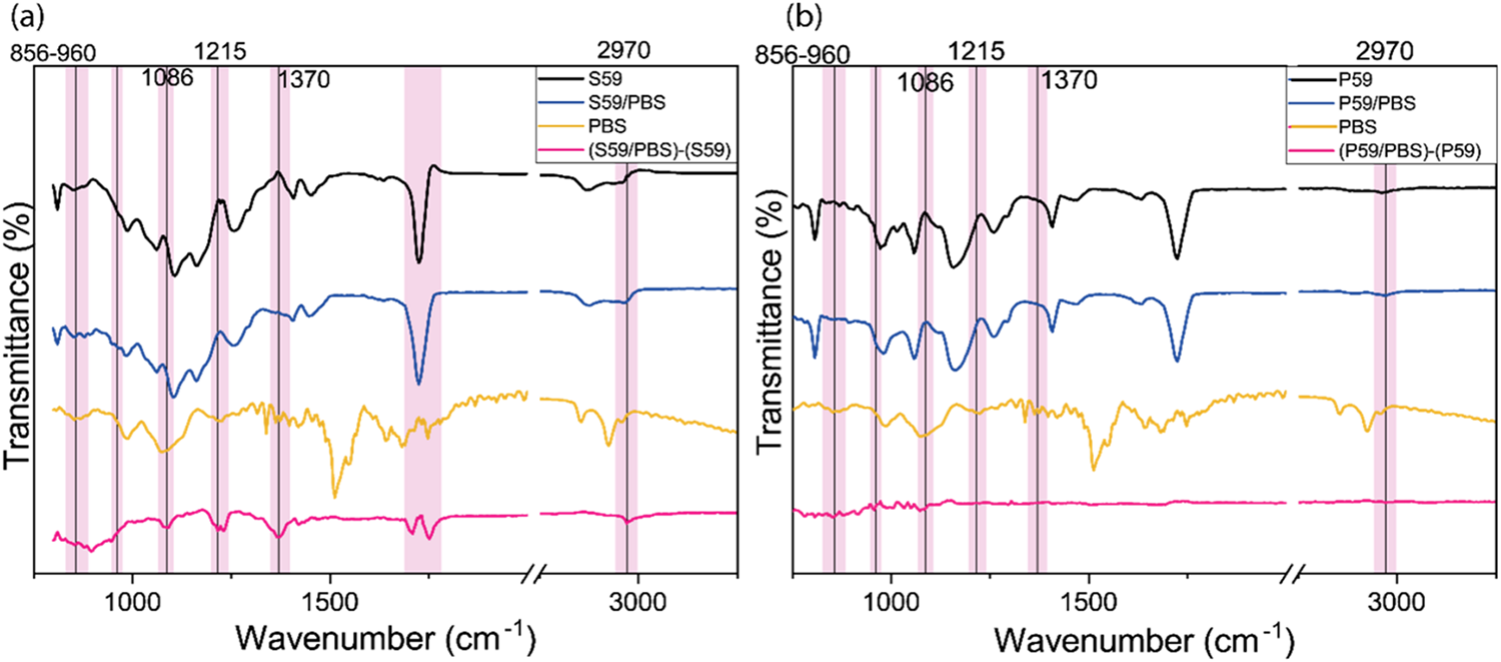
FTIR characterization spectra of the samples (a) S59, (b) P59 and difference spectrum of the polymeric films (original film minus PBS-treated film).

In the spectra, phosphate bands attributed to the PBS treatment were observed. However, additional bands were detected for the S59 substrate, indicating interactions with PBS, mainly the formation of oxygenated and hydroxylated groups, such as OH bending at 1418 cm^–1.^ The peaks at 1760 cm^–1^ and 1714 cm^–1^ correspond to the formation of alkyl carbonate and carboxylic acid functional groups, respectively. The peak at 1232 cm^–1^ is attributed to the stretching vibrations of aryl-O. The peak at 2972 cm^–1^ is related to methylene. Notably, the vibrational modes centered at 1370 cm^–1^ (hydroxyl bond (−OH)) and 1086 cm^–1^ (P–O stretching of the phosphate ion), the latter specifically associated with H_2_PO_4_^–^ or HP*O*_4_^2–^ bonding, suggest the formation of a thin phosphate layer on the S59 surface after the adsorption process.

Figure 9a shows the values of the measured morphological parameters for a better analysis of cell adhesion to surfaces treated with PBS. For the CTRL samples, the cells exhibited an elongated shape with a larger cell area, resulting in a Shape Factor and aspect ratio of 0.56 ± 0.06 and 0.32 ± 0.07, respectively, which indicates high cell adhesion.

**Figure 9:**
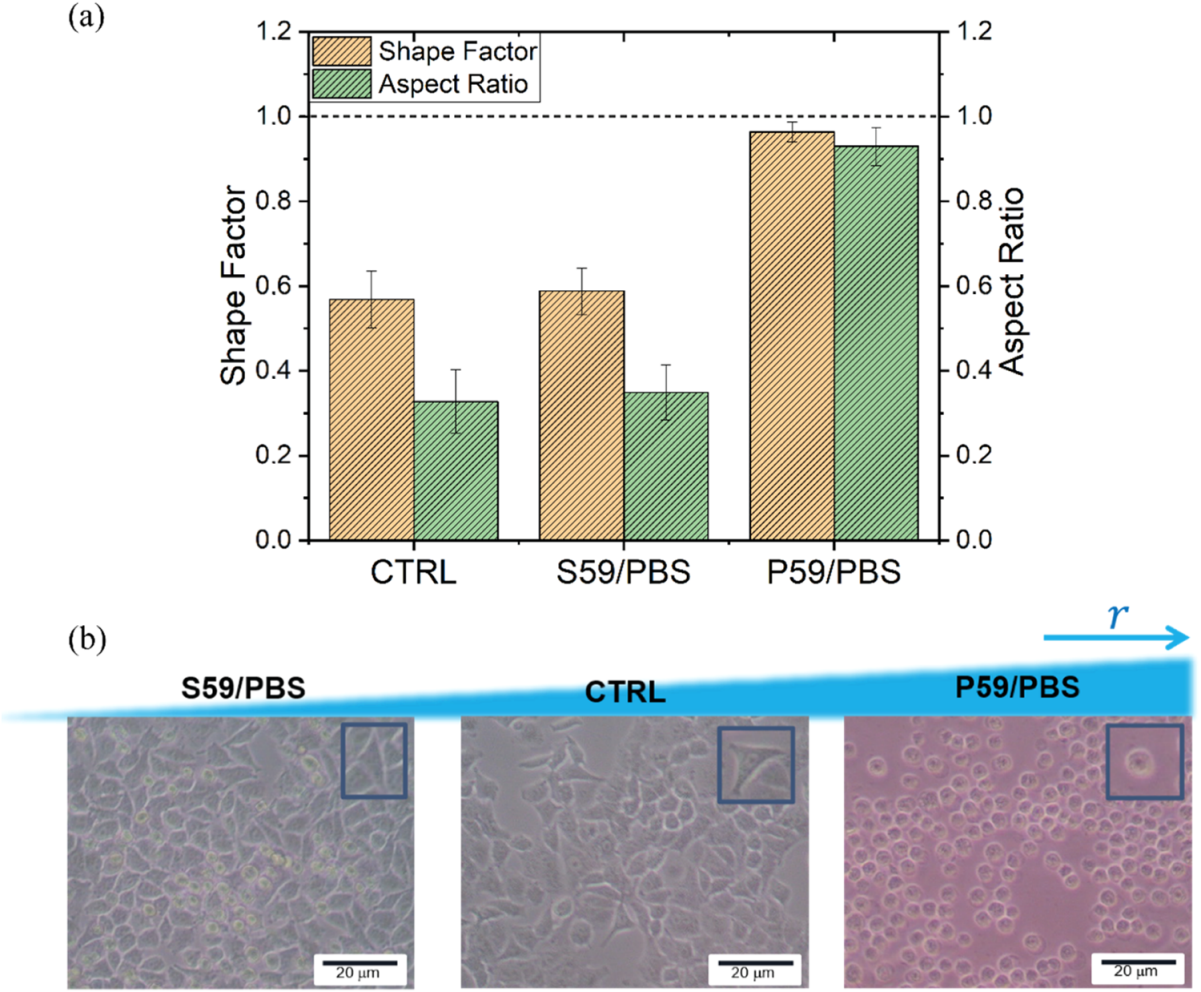
(a) The values of the Shape Factor and Aspect Ratio of A549 cells after surface modification with PBS and CTRL. (b) Experimental images of A549 cells cultured on different surfaces for 24 h.

Our analysis showed that the PBS treatment of the S59 substrate increased the adhesion of A549 cells. The Shape Factor and aspect ratio values for these samples were 0.58 ± 0.05 and 0.34 ± 0.06, respectively, the closest to the control. For the P59/PBS samples, the values of these parameters were closer to 1, indicating lower cell adhesion to the surface. The cell behavior quantified and presented in Figure 4a can be further observed in Figure 9b through the analysis of cell morphology. We can clearly see how the S59 substrate promotes both cell adhesion, similar to the CTRL plates, and cell proliferation, like the P59 substrates—two essential cellular behaviors when developing biomaterials for tissue engineering.

## 4 Discussion

The cell–substrate interaction is strongly influenced by physicochemical properties, including wettability, roughness, and surface free energy. Consequently, controlling these characteristics allows predicting and improving biological activities, including cell adhesion. Polymers have been widely used as substrates for 2D cell cultivation and in the fabrication of 3D cell scaffolds.^43–45^ Although acrylic-based polymers have numerous advantages, they are materials with physicochemical characteristics that can inhibit cell adhesion.^46,47^ Since each cell type has specific adhesion properties, it is necessary to adjust the parameters present in the substrate to match the cell behavior, providing adequate conditions for spreading.

Previous studies have already investigated the modification of the polymeric surface roughness by the PBS buffer together with thermal treatment.^48^ Unlike previous studies, here we performed the treatment with PBS on polymeric surfaces S59 and P59, demonstrating its effect without the need for additional heat treatment, and it had a dominant effect on the adhesion of pulmonary epithelial cells. This occurred because the physicochemical properties related to wettability, surface energy, and roughness ratio are modified after the interaction with PBS, which are characteristics already known to influence cell morphology and adhesion.^23,49,50^

In our *in vitro* studies to determine cell adhesion, we demonstrated that the cells showed higher adhesion and elongation after 24 h on the S59/PBS substrate. Under this condition, the values of the shape factor and the cellular aspect ratio were closer to the control (S59/PBS: 0.56 and 0.32; CTRL: 0.58 and 0.34, respectively), reflecting the morphological confirmation of the cells. However, for the P59/PBS samples, the shape factor and the cellular aspect ratio values approached 1 (0.96 and 0.92, respectively), resulting in less cell adhesion to the material’s surface.

The mechanical stiffness of the polymeric matrix plays an important role in modulating cellular functions, including the degree of cell–substrate adhesion.^51^ The S59 polymers exhibited a significantly lower elastic modulus and greater elongation at break compared to P59, indicating polymeric matrices that are less rigid and more flexible. Studies have shown that less rigid substrates may promote the migration of A549 pulmonary epithelial cells,^52^ which supports our evidence that the best cell adhesion performance was observed in polymers with a lower elastic modulus (S59). Furthermore, the elastic modulus values obtained indicate that the substrates possess mechanical properties compatible with biomedical applications.^53^

From there, we verified how these morphological changes were related to the roughness ratio on the polymer surfaces. Indeed, this is a crucial parameter to determine the effectiveness of cell adhesion, as it describes the actual interaction between the cell and the rough surface.^54^ Once again, S59/PBS and CTRL coincided and presented a moderate roughness ratio (1.005 ≤ *r* ≤ 1.008), allowing the cell cytoplasmic membrane to conform to the grooves of the rough surface, promoting maximum interaction and stability between the cell and substrate, resulting in better adhesion. On the other hand, P59/PBS presented a higher roughness ratio (*r* ≥ 1.014), making it difficult for the cell cytoplasmic membrane to adapt to irregularities and fully reach all surface grooves. As a result, the cells were located on top of the rough substrate’s protrusions without touching the bottom of the grooves. This reduced contact interaction between cell and substrate led to a significant decrease in cell adhesion, a phenomenon that finds support in previous works.^24^

S59/PBS exhibited a combination of strongly negative skewness, extremely high kurtosis, and low average height, indicating a surface with deep valleys and sharp topographical features that may create favorable microenvironments for protein adsorption and cell anchoring. This structural complexity, together with surface functionalization, is consistent with the high cell adhesion observed, comparable to the control. In contrast, P59/PBS showed a more uniform topography, which may have limited cell–substrate interactions despite increased wettability.

It is important to mention that the migratory behavior of cells is strongly influenced by the constituents of the extracellular matrix (ECM). With this, we can speculate that the S59 and P59 polymers could induce the production of different ECM proteins that would result in distinct migratory behavior as well. Future studies may be conducted to better understand this point.

Furthermore, through FTIR analysis, we demonstrated that the PBS treatment was effective in functionalizing the polymeric substrate S59. The FTIR spectrum of these samples showed the presence of hydroxyl (OH) and carboxylic acid (COOH) functional groups, essential for promoting cell adhesion. Several studies focused on the biological activity of polymers indicate that the carboxyl and hydroxyl groups, along with the surface roughness promoted in the substrate, have the potential to promote excellent cellular affinity.^21,55–57^

Also, it is already known that adjusting the surface free energy can influence cell adhesion.^58,59^ Taking as a reference a commercially treated polymeric substrate to improve cell adhesion with a surface free energy of 42.78 mJ/m^2^, we observed that the S59/PBS substrates approached their surface free energy to that of the control material (57.0 mJ/m^2^), which did not occur with the P59/PBS substrate (63.44 mJ/m^2^). This result is strongly linked to the fact that surface energy controls the material’s wettability, which, in turn, can affect the number of proteins adsorbed during the interaction of cells and the polymeric substrate.^60,61^ While all the results obtained in this study are clear, it is important to note that FTIR analyses can have limitations, particularly when detecting compounds at low concentrations (less than 5%). Therefore, we intend to expand our analyses in future studies using other techniques, such as XPS and ToF-SIMS, which offer better sensitivity to provide more detailed chemical information on the surface after PBS treatment.

Plasma treatment is a well-established method for polymer surface modification, capable of introducing polar functional groups and altering surface topography at micro- and nanoscale levels, thereby significantly enhancing hydrophilicity and cell adhesion.^55,62^ However, it typically requires specialized equipment, controlled operational parameters, and can involve high costs and energy consumption. In contrast, PBS treatment, as investigated in our study, provides a simpler, cost-effective, and environmentally friendly alternative that does not demand complex infrastructure or hazardous chemicals. Our results show that the moderate roughness and increased polar groups achieved with PBS are sufficient to improve cell adhesion under the tested conditions.

Tissue engineering strategies and biomedical applications should align with sustainability goals to minimize carbon footprint.^63^ Conventional biomaterials and surface modification processes often rely on energy-intensive processes and nonrenewable resources, increasing greenhouse gas emissions. In contrast, simple surface modifications, such as phosphate-buffered saline treatments, offer a low-impact approach that avoids harsh chemicals and high energy consumption. Incorporating such eco-friendly methods supports both advanced tissue engineering and global efforts to mitigate climate change.

These results indicate that we can design and develop 3D cell scaffolds and further adjust the environmental conditions through PBS treatment to promote cell adhesion. This optimization enhances the microfabrication process and facilitates the study of cellular behavior, as we can follow the basic protocols of biological assays, namely: scaffold fabrication through two-photon polymerization, removal of nonpolymerized resin, treatment with PBS, sterilization, and cell culture.

## 5 Conclusions

The effect of PBS treatment as a method for modifying the surface properties of polymeric films made from S59 presents unique advantages studied here, such as moderate roughness, increased hydrophilicity, and the emergence of functional groups. This makes it an excellent candidate for controlling cell functions and tissue regeneration. The findings of this study open up new possibilities for including PBS treatment as a surface adaptation method to regulate cell responses, among the various surface treatment techniques already known, due to its being an extremely simple, fast, eco-friendly, low-cost, and efficient process with a green footprint. The tendency of PBS treatment to improve the adhesion of A549 pulmonary epithelial cells to the S59 polymer is of significant relevance, as it allows for the applicability of polymeric materials that were previously unsuitable for cell cultivation, as they did not enable the cells to exhibit their cellular behavior. Our study and conclusions were based on optical observations, spectroscopic characterizations, and mechanical analyses involving the cells and the substrate surface. Our results demonstrate that it is possible to regulate the physicochemical properties of the polymeric substrate with PBS treatment to enhance cellular responses.

## Data Availability

All data generated or analyzed during this study are included in this published article.
